# Small volume plasma exchange for Guillain-Barré syndrome in resource poor settings: a safety and feasibility study

**DOI:** 10.1186/s40814-017-0185-0

**Published:** 2017-09-29

**Authors:** Md. Badrul Islam, Zhahirul Islam, Shafiqur Rahman, Hubert P. Endtz, Margreet C. Vos, Mathieu van der Jagt, Pieter A. van Doorn, Bart C. Jacobs, Quazi D. Mohammad

**Affiliations:** 1 Department of Medical Microbiology and Infectious Diseases, Erasmus University Medical Center, Rotterdam, The Netherlands; 2 Laboratory Sciences and Services Division (LSSD), International Centre for Diarrheal Disease Research, Bangladesh (icddr,b), Dhaka, Bangladesh; 3 Department of Intensive Care Medicine, Uttara Adhunik Medical College & Hospital, Dhaka, Bangladesh; 4 Fondation Mérieux, Lyon, France; 5 Department of Intensive Care, Erasmus University Medical Center, Rotterdam, The Netherlands; 6 Department of Neurology, Erasmus University Medical Center, Rotterdam, The Netherlands; 7 Departments of Neurology and Immunology, Erasmus University Medical Center, Rotterdam, The Netherlands; 8 National Institute of Neurosciences & Hospital, Dhaka, Bangladesh

**Keywords:** Guillain-Barré syndrome, SVPE, IVIG, PE, ICU, HDU, Safety, Feasibility, SAE

## Abstract

**Background:**

In Bangladesh, most patients with Guillain-Barré syndrome (GBS) cannot afford standard treatment with intravenous immunoglobulin (IVIG) or a standard plasma exchange (PE) course, which partly explains the high rate of mortality and residual disability associated with GBS in this country. Small volume plasma exchange (SVPE) is an affordable and potentially effective alternative form of plasma exchange. SVPE is the repeated removal of small volumes of supernatant plasma over several days via sedimentation of patient whole blood. The aim of this study is to define the clinical feasibility and safety of SVPE in patients with GBS in resource poor settings.

**Methods:**

A total of 20 adult patients with GBS will be enrolled for SVPE at a single center in Bangladesh. Six daily sessions of whole blood sedimentation and plasma removal will be performed in all patients with GBS with a target to remove an overall volume of at least 8 liters (L) of plasma over a total of 8 days. Serious adverse events (SAE) are defined as the number of patients developing severe sepsis associated with the central venous catheter or deep venous thrombosis in the limb where the catheter is placed for SVPE. Based upon a predictive success rate of 75%, the SVPE procedure will be considered safe if less than 5 of 20 SVPE-treated GBS patients have a SAE. The procedure will be considered feasible if 8 L of plasma can be removed in at least 15 of 20 patients with GBS who receive SVPE. In addition, detailed clinical and neurological outcome assessments will be performed until discharge of the patient from the hospital and up to 4 weeks after study entry.

**Discussion:**

This is the first clinical study to evaluate the feasibility and safety of SVPE as a potential alternative low-cost treatment for the patients with GBS in resource poor settings.

**Trial registration:**

Clinicaltrials.gov NCT02780570

**Electronic supplementary material:**

The online version of this article (10.1186/s40814-017-0185-0) contains supplementary material, which is available to authorized users.

## Background

Guillain-Barré syndrome (GBS) is an acute poly-radiculo-neuropathy with a yearly incidence of 1.2 to 2.3 per 100,000 [1]. GBS is characterized by rapidly progressive symmetrical weakness of the limbs that reaches a plateau within 4 weeks. Recovery usually starts after a plateau of 2 to 4 weeks but may be delayed for months [2]. In addition, some patients experience sensory, cranial, or autonomic nerve deficits or respiratory failure. GBS is highly variable with respect to its clinical and electrophysiological presentation and differs between patients from the Eastern and Western hemisphere [3–5]. Acute inflammatory demyelinating polyneuropathy (AIDP) [3, 4] with sensory-motor involvement is the predominant form in the Western world whereas most patients in Asian countries have the acute motor axonal neuropathy (AMAN) form. At present two treatment interventions are used, that hasten the recovery of GBS. Plasma exchange (PE) was the first treatment proven to be effective in GBS, if given within 4 weeks of the onset of weakness [2, 3, 6–12]. Four sessions of standard PE (50 mL/kg plasma per session) in exchange with fresh frozen plasma (FFP) or albumin-saline mixture is beneficial in patients with GBS who are unable to walk at nadir [7, 13–16]. Efficacy of intravenous immunoglobulin (IVIG) (0.4 g/kg per day for 5 days) is similar to PE if given within 2 weeks of the onset of weakness in patients with GBS who are unable to walk [17, 18].

In low-income countries, most patients cannot afford treatment with either PE or IVIG [19]. In Bangladesh, a full course of IVIG for a 60-kg adult costs approximately 12,000–16,000 USD, and treatment with conventional PE for 5 days costs approximately 4500–5000 USD. As a result, only 15% of patients with GBS in Bangladesh are treated with IVIG or PE, while the remaining 85% of patients only receive supportive care. Patients with GBS in Bangladesh have a poor prognosis compared to patients in Western countries. We previously reported a mortality rate of 14% and observed 29% of patients with GBS in Bangladesh are unable to walk 6 months after disease onset, which may in part be due to the low rates of specific treatment with PE or IVIG [5].

Small volume plasma exchange (SVPE) may represent an effective alternative treatment for GBS. SVPE is based on the same principle as conventional PE but uses a novel but simple technique and has a much lower cost (approximately 500 USD). SVPE is achieved by repeated removal of small volumes of supernatant plasma over several days via sedimentation of patient whole blood. This study was designed to investigate the safety and feasibility of SVPE in 20 patients with GBS admitted to the National Institute of Neurosciences and Hospital in Dhaka, Bangladesh.

### Specific objectives

The objective of this study is to determine the safety and feasibility of SVPE in patients with GBS in Bangladesh.

## Methods/design

### Study design

**Fig. 1:**
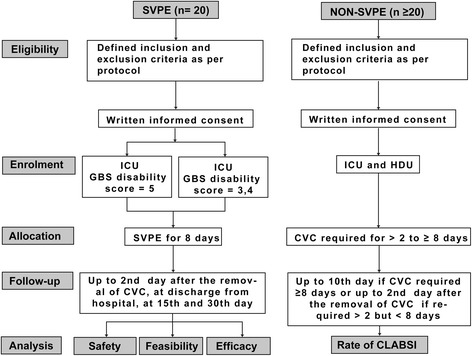
Consolidated Standards of Reporting Trials (CONSORT) diagram for the small volume plasma exchange (SVPE) safety and feasibility study

In this safety and feasibility study, 20 adult patients with GBS will be enrolled for SVPE at the National Institute of Neurosciences and Hospital, Dhaka, Bangladesh. Patients will be monitored according to a standard protocol throughout the course of SVPE until the second day after withdrawal of the central venous catheter (CVC) to assess predefined measures of feasibility, safety, and neurological recovery. To compare the safety of SVPE in patients with GBS in the context of the background risk of central line-associated blood stream infection (CLABSI) at our institution, we will assess the incidence of CLABSI in a control group of adult patients with a diagnosis other than GBS admitted to the same intensive care (ICU) and high-dependency care (HDU) units in the same period of time (Fig. 1 and Table 1).

Patient recruitment began in March 2016 and is expected to last for 18 months. Final analysis and preparation of manuscript will be done within 3 months after the last patient with GBS completes the SVPE.

### Ethical consideration

The protocol was reviewed and approved by the Institutional Review Board (IRB) consisted with Research Review Committee (RRC) and the Ethical Review Committee (ERC) at icddr,b (Protocol Number: PR-15086, Version no. 3, Date: December 9, 2015). The study will be performed according to the Declaration of Helsinki [20]. Only patients who provide informed signed consent to the study physician would be included in the study. The participants will be informed that participation in the trial is voluntary and that they can withdraw consent at any time without giving reasons. Expenditure for the patients with GBS treated with SVPE and any harm related to the SVPE will be fully compensated.

### Patient enrolment—case definition

#### Patients with GBS

Patients ≥ 18-years of age fulfilling the diagnostic criteria for GBS of the National Institute of Neurological and Communicative Disorders and Stroke (NINDS) [21] will be enrolled, provided they are unable to walk unaided for more than 10 meters (GBS disability score ≥ 3), presented within 2 weeks of the onset of weakness, and are unable to afford standard treatment with IVIG or PE. Patients with severe or terminal concomitant illness, evidence of healthcare-associated infection on admission (except for aspiration pneumonia), or a previous history of severe allergic reaction to properly matched blood products and pregnant women will be excluded from the study.

#### Patients without GBS

Patients ≥ 18-years of age with a diagnosis other than GBS requiring a CVC for > 2 to ≤ 8 calendar days after admission to the same ICU and HDU units in the same period of time will be eligible. Patients with healthcare-associated infection present on admission (except aspiration pneumonia) and pregnant women will be excluded from the control group.

### Primary outcome measures

The primary outcome measures of safety are the number of patients with GBS treated with SVPE developing severe sepsis or septic shock (Additional file 1 for standardized definitions: Appendix 1A) due to CLABSI [22] and the occurrence of venous thrombosis in the limb where the CVC is placed (Additional file 1 for standardized definitions: Appendix 1B). The primary outcome measure of feasibility is the ability to remove at least 8 L of plasma over 8 days.

### Secondary outcome measures

The secondary outcome measures of safety of SVPE are the relative risk of CLABSI due to SVPE compared to CLABSI in control patients without GBS treated using a CVC, hemodynamic instability during the SVPE procedure (variations in systolic blood pressure greater than 30 mmHg or sudden bradycardia involving a reduction in heart rate by more than 20 beats per min within 30 min of starting SVPE or an increase in heart rate above 120 beats per min), and development of anemia (Hb < 8 g/dL) or serious hemorrhage requiring blood transfusion.

The secondary outcome measures of the feasibility of SVPE are the rate of CVC occlusion during the SVPE procedure, the healthcare personnel’s acceptability and satisfaction with the SVPE procedure, and any unanticipated events compromising the SVPE procedure as assessed using a standard questionnaire (Additional file 1 for standardized definitions: Appendix 1C). In addition, neurological outcome will be assessed in terms of improvement in GBS disability score [23] and MRC sum score [24] at discharge and up to 4 weeks after entry.

### Attempts to minimize the risks associated with SVPE

**Fig. 2:**
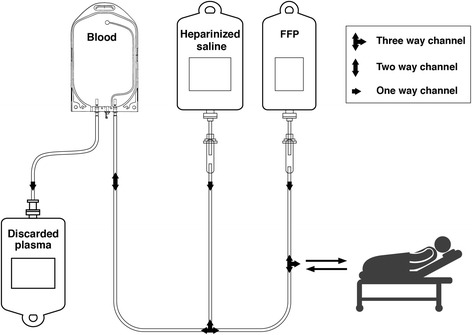
Illustration of the small volume plasma exchange (SVPE) kit. A SVPE kit will be prepared with one blood transfusion set and two saline infusion sets connected via a tri-channel device. The blood transfusion set will be connected to the blood bag and a saline infusion set will be connected to 1 L normal saline mixed with 2500 units of unfractionated heparin and the other ends of the blood transfusion and saline infusion sets will be connected with one lumen of the central venous catheter using a tri-channel device. Another saline infusion set will be connected to 500 mL hexa-ethyl starch solution and the other end connected with the second lumen of the central venous catheter. Air should be eliminated from all tubes using fluid from the respective bags

All connection ports of the SVPE kit (Fig. 2), the lumens of the CVC, the blood bag, and the saline bag will be disinfected with 70% alcohol and then air-dried before sampling blood or disconnection. Strict aseptic procedures will be followed to prevent CLABSI [25–27]. The SVPE procedure will not be started or stopped temporarily after the start of SVPE in patients who develop hypotension (30 mmHg decrease in systolic blood pressure, systolic blood pressure < 90, or MAP < 65 mmHg) or cardiac arrhythmia for any reason. After correction of hypotension and/or cardiac arrhythmia, the SVPE procedure will be started or restarted if SVPE stopped due to autonomic instability. The CVC will be removed as soon as SVPE is completed or the CVC will be immediately relocated to another insertion site if a CLABSI related to the CVC used for SVPE is confirmed or strongly suspected. Patients with GBS having severe sepsis as per standardized definition (Additional file 1: Appendix 1A) due to any organ site-specific infection present on admission (except for aspiration pneumonia) will be excluded for SVPE. Otherwise, any covert infection or aspiration pneumonia, as may be indicated by leukocytosis, will be investigated and treated accordingly after consultation with a medical microbiologist. Normal hemoglobin concentration for > 18 years man and non-pregnant woman will be considered as 13 and 12 g/dL, respectively [28]. However, blood transfusion will be considered to correct severe anemia (hemoglobin level < 8 g/dL [28]) before, during, or after SVPE. Proper screening of FFP for specific infective agents (hepatitis B and C viruses, human immunodeficiency virus (HIV), and syphilis) will be performed prior to administration of FFP.

### Documentation (Table 1)

#### Baseline assessment and re-evaluation

Complete blood counts, including hemoglobin (g/L), erythrocyte sedimentation rate (ESR), C-reactive protein, and coagulation profile as well as serum calcium, magnesium, albumin, creatinine, bilirubin, and SGPT will be documented as baseline assessments for patients with GBS treated with SVPE, along with screening for hepatitis B, C, and HIV. Baseline assessments will be re-evaluated on day 4 (interim analysis) and day 10 (second day after completion of SVPE and removal of the CVC) after initiation of SVPE.

**Table 1:** Standard Protocol Items: Recommendations for Interventional Trials (SPIRIT) table for the SVPE safety and feasibility study

	Study period							
	Enrolment	Allocation	Post-allocation					Close-out
	Entry^a^	0	Day 1–3	Day 4	Day 5–8	Day 10	Discharge*	Day 30*
Time point								
Eligibility screen	+							
Informed consent	+							
Allocation		+						
Interventions								
SVPE			+	+	+			
Non-SVPE			+	+	+			
Assessments								
SVPE procedure^b^			+	+	+			
Hemodynamic^c^	+	+	+	+	+	+	+	
Biochemistry^d^	+	±‡	±‡	+	±‡	+		
Infection screening^e^	+	±‡	±‡	+	±‡	+		+
Neurological^f^	+	+		+		+	+	+

*For patients treated with SVPE. ‡ Assessed as per indication

^a^Within 14 days of onset of muscle weakness

^b^Number and duration of each SVPE session and volume of the plasma removed and the replacement fluid infused

^c^Blood pressure, heart rate, body temperature, and pulse oximetry

^d^Hemoglobin (g/L), coagulation profile, serum level of calcium, magnesium, albumin, renal, and liver function test

^e^Complete blood count, erythrocyte sedimentation rate (ESR), C-reactive protein, and microbiological investigations to assess CLABSI, CAUTI, and VAP

^f^GBS disability score and MRC sum score

#### The procedure, hemodynamic parameters, and cost for SVPE

**Fig. 3:**
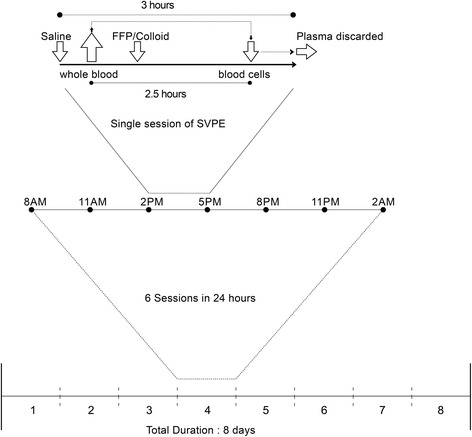
SVPE procedure for patients with GBS. A loading dose of low-molecular weight heparin (1.5 mg/kg) will be given subcutaneously at least 2 hours before initiation of SVPE; the same dose will be administered once daily or divided into two equal doses daily for 8 days or until SVPE is completed. Whole blood (7 mL/kg body weight) will be drawn from the central venous catheter into the blood transfusion bag in each session. The blood bag will be hung beside the patient for 2.5 h on a saline stand and left uninterrupted to allow plasma and blood cells to separate. The blood cells will be infused back into the patient and plasma will be discarded and replaced with fresh frozen plasma and colloid solution alternately (in equal volumes) via the closed-circuit SVPE kit illustrated in Fig. 2. In case of excessive clotting (bleeding time reduction of > 50% of baseline for that patient), aspirin (600 mg) will be administered orally at least 2 hours before the next SVPE session and continued thereafter at 150 mg orally/day until SVPE is completed. One blood bag will be used each day, with a total of six sessions/day. A total of 48 sessions will be performed over 8 days, removing approximately 8000 mL plasma in total

SVPE will be documented in terms of the duration and amount of plasma removed in each session, the type and volume of replacement fluid, and FFP used (Fig. 3). Throughout the procedure, hemodynamic status including blood pressure, heart rate, any cardiac arrhythmia, oxygen (%) saturation in peripheral blood, and fluid intake and output will be monitored daily according to the protocol. Autonomic parameters especially the presence of high or low blood pressure, increased or decreased pulse rate, and cardiac arrhythmia will be monitored and treated accordingly, before and after each SVPE session (12 times a day) and if necessary during and in between the SVPE sessions. Bladder and gastrointestinal dysfunction, pupillary abnormality, unusual sweating, and hyper-salivation will also be monitored daily. Any inconvenience experienced by the patient, nurse, or physician that temporarily or permanently compromise the SVPE sessions (Additional file 1 for standardized definitions: Appendix 1C). The cost for the SVPE in total 8 days will be approximately 500 US$ [fresh frozen plasma (24 bags) = 240 US$, blood bag and saline sets: 40 US$, low molecular weight heparin: 110 US$, routine investigation: 50 US$, saline: 10 US$, CV catheter: 40 US$ = total 490 US$].

#### The hospital-acquired infections (HAI) and complications

CLABSI and primary and secondary bloodstream infections [22], catheter-associated urinary tract infection (CAUTI) [29], ventilator-associated pneumonia (VAP) [30], and other HAIs [31, 32] will be stringently documented in SVPE treated patients with GBS and non-GBS baseline control patients requiring the CVC in the ICU/HDU area using predefined criteria. Sepsis and severe sepsis will be documented as per protocol definition (Additional file 1 for standardized definitions: Appendix 1A). All cases of definite or strongly suspected CLABSI related to SVPE will be followed up until discharge to document the severity of CLABSI. Appropriate clinical data, imaging, and biochemical and microscopic analyses of specimens and bacteriological cultures will be documented as per indication of site-specific infections. Device-associated complications including CVC occlusion, hemorrhage, and deep vein thrombosis [33] will be documented in real-time.

#### Neurological function

Neurological examinations will be performed to monitor the clinical changes in motor, sensory, and autonomic parameters before and during the procedure and in the subsequent follow-up of patients lasting up to 4 weeks. Disability and motor function will be assessed using the GBS disability score [23] and MRC sum score [24]. Somatic and proprioceptive sensation of the limbs will be assessed bilaterally.

### Sample size

This safety and feasibility study will enroll 20 patients with GBS for SVPE. We cannot perform a formal power calculation for this safety and feasibility study. The sample size is based on previous pilot studies in GBS [34, 35]. The baseline rate of CLABSI will be measured in parallel in patients without GBS (and not undergoing SVPE) admitted to the same study facility who require a CVC during the study period. All control patients available during the inclusion time period will be included. We expect to obtain at least 20 or more (i.e., the maximum number of patients possible during the study period) control patients without GBS who require a CVC for at least 2 days and maximum of 8 days.

### Indication to stop the trial

Decision to stop the SVPE trial will be designated as per a Bayesian approach [36–38]. Accordingly, a predictive success rate of 75% will be set for the SVPE procedure. If more than 5 of 20 patients experience an SAE or when it appears to be impossible to remove at least 8 L of plasma in at least 15 individual patients, the procedure will be considered unsafe or unfeasible, respectively.

### Data management

The unique patient identity (ID), name, date of birth, and basic demographic and clinical data of the SVPE-treated patients and control patients will be stored separately in paper and electronic database documentation and updated on a daily basis. All the privacy-sensitive data will be stored encrypted. In case of withdrawal, follow-up and data collection will continue with the participant’s permission. A data safety monitoring board (DSMB) consisting of clinical experts, a microbiologist, and a biostatistician from the icddr,b ERC committee will be responsible for the monitoring and review of the safety and conduct of this safety and feasibility study. The DSMB will function independently and has the right to audit the study without notice to the investigators and the sponsor. Important protocol amendments for approval and AEs that occur at a greater frequency or intensity than expected will be reported to the IRB of the icddr,b within 7 business days. However, any AE without any discretion will be reported to the DSMB and the ERC within 24 h. Upon completion of the trial, all study-related data and trial documents will be archived securely at the icddr,b and retained for a minimum of 10 years. The corresponding author will have full access to all the data in the study and will be responsible for the decision to submit the publication.

### Statistical analysis

The rate of HAIs (CLABSI, CAUTI, VAP) per 1000 device days will be calculated by dividing the number of HAIs (CLABSI, CAUTI, VAP) during the study period by the number of device days and multiplying the result by 1000. The infection safety profile for SVPE will be assessed by calculating the standardized infection ratio (SIR) to define the risk of HAI in patients with GBS treated with SVPE. The SIR will be calculated by dividing the number of observed infections by the number of infections predicted (i.e., the infection rate in the control group). The predicted HAI rate will be calculated using the rates of HAI in the control group of patients without GBS during the study period. Percentage values will be compared using chi-square test or Fisher’s exact test (two-tailed) and median values, the Mann-Whitney *U* test using SPSS 22 software (IBM Corp. Released 2013. IBM SPSS Statistics for Windows, Version 22.0. Armonk, NY: IBM Corp). Analyses will be performed on an intention-to-treat basis. All *p* values reported will be two sided, and a difference of *p* < 0.05 will be considered significant*.*

## Discussion

SVPE is based on the removal of supernatant plasma after sedimentation of whole blood and represents a putative alternative treatment for GBS in low-income countries where most patients cannot afford PE or IVIG [19]. In countries like Bangladesh, a safe, effective treatment needs to be available at local hospitals so patients can obtain medical care as soon as possible. Considering the current infrastructure and high costs of specific treatments, access to IVIG or standard PE remains a challenge for many patients with GBS in Bangladesh and other low-resource developing countries.

Compared to conventional PE (four sessions at a rate of 50 mL/kg plasma removal per session [7, 13–16]; approximate removal of 12 L of patient plasma in total), a slightly lower volume of plasma is exchanged during SVPE, though this volume is reached by daily exchange of small volumes of plasma (approximately 1 L/day). The potential advantages of SVPE are its simplicity and low cost (approximately 500 USD) and the fact that it does not require advanced equipment and can be performed over 8 days compared to standard PE, which requires a total period of 10 to 14 days. Before assessing the potential efficacy of SVPE for GBS, it is essential to define the feasibility and safety of this therapeutic intervention in local hospitals.

SVPE is time-consuming, and the ability to complete uninterrupted treatment could be hampered due to autonomic dysfunction. Considering these issues, we predefined the criterion that SVPE will be considered feasible if at least 8 L of plasma can safely be removed in 8 days. Infections related to the CVC are the major safety concern during SVPE. The rate of HAI may be much higher in low-income countries, especially in the Southeast Asia, compared to high-income countries. According to the International Nosocomial Infection Control Consortium (INICC), the rates of CLABSI are significantly higher in ICUs in low-income countries (6.8 per 1000 central line-days) than the US (2.0 per 1000 central line-days) [39]. Additionally, the microbiologic spectrum varies, with a higher tendency for gram-negative bacteria to cause CLABSI in developing countries [40–43] as opposed to gram-positive bacteria in developed countries [27]. Poor infrastructure, inadequate hygienic maneuvering of vascular catheters, and a lack of knowledge regarding CVC safety may contribute to the increased risk of CLABSI in developing countries. Additionally, the indiscriminate use of broad-spectrum antibiotics further escalates multi-drug resistance.

No data is available on the prevalence of CLABSI within ICUs or HDUs in Bangladesh. Therefore, this study will systematically document the rate of CLABSI in the ICU/HDU of the institution, at which this study is being conducted, with the aim of comparing the baseline risk of infection with the risk of CLABSI associated with SVPE. Implementation of the protocol-defined safe techniques for vascular catheter handling and maintenance could improve the knowledge and awareness of the health personnel at the study facility. Eventually, a positive impact on safety is expected in terms of infection control. Though SVPE is a simple procedure, the motivation of and coordination between doctors and nurses is critical to the feasibility of this technique. Therefore, we decided to employ a questionnaire-based qualitative approach to assess the experiences of the participating medical personnel with the SVPE procedure. However, a drawback is the fact that we did not assess psychometric properties of this questionnaire in the study settings. However, our consideration is to generate some evidence within the study.

Continuous wide-lumen tubing with built-in keys to direct blood/saline flow reduces manipulation and may reduce the chance of contamination. However, these systems are currently unavailable in Bangladesh; therefore, we employed multiple thin-lumen tubing systems interconnected with a multichannel connecter device.

Important advantages of SVPE over standard PE are the low cost and ability to perform SVPE by the bedside of the patient. In most hospitals in Bangladesh, patients requiring mechanical ventilation cannot be transferred to the PE facility, as there is no facility for mobile PE. Moreover, SVPE could represent a therapeutic option in resource-limited settings that lack the facility for PE. Should the SVPE procedure be feasible and safe, we plan to conduct a randomized control trial in the future to determine the clinical efficacy of SVPE for the treatment of GBS in developing countries as an alternative to conventional treatment with IVIG or PE.

### Recruitment status

Recruitment has been completed with 20 cases of GBS treated with SVPE and 24 control cases without GBS in June 2017.

## Additional file

Additional file 1: Standardized definitions. (DOCX 85 kb)

## Abbreviations

AE: Adverse effect
AIDP: Acute inflammatory demyelinating polyneuropathy
AMAN: Acute motor axonal neuropathy
CAUTI: Catheter-associated urinary tract infection
CDC: Communicable Disease Control
CLABSI: Central line-associated blood stream infection
CRF: Case record form
CVC: Central venous catheter
DSMP: Data safety monitoring plan
DVT: Deep vein thrombosis
ERC: Ethical Review Committee
FFP: Fresh frozen plasma
GBS: Guillain-Barré syndrome
HAI: Healthcare-associated infection
HBV: Hepatitis B virus
HCV: Hepatitis C virus
HDU: High-dependency unit
HIV: Human immunodeficiency virus
ICU: Intensive care unit
IRB: Institutional review board
IVIG: Intravenous immunoglobulin
MAP: Mean arterial pressure
MRC: Medical Research Council
NCS: Nerve conduction study
NHSN: National Healthcare Safety Network
NINDS: National Institute of Neurological Disorders and Stroke
ONLS: Overall neuropathy limitation scale
PE: Plasma exchange
PICC: Peripherally inserted central catheter
RCT: Randomized control trial
R-ODS: Rasch-built overall disability score
RRC: Research Review Committee
SAE: Severe adverse effect
SIR: Standardized infection ratio
SVPE: Small volume plasma exchange
VAE: Ventilator-associated event
VDRL: Venereal Disease Research Laboratory
