# Supplementary material for: Small volume plasma exchange for Guillain-Barré syndrome in resource poor settings: a safety and feasibility study
Source: Pilot Feasibility Stud. 2017 Sep 29;3:40. doi: 10.1186/s40814-017-0185-0 (PMC5622586; doi:10.1186/s40814-017-0185-0)
Supplement: Additional file 1: — Standardized definitions. (DOCX 85 kb) [file 40814_2017_185_MOESM1_ESM.docx]

**APPENDIX 1A**

**Infection, bacteraemia, SIRS, sepsis, septic shock**

**Infection:**

Microbial phenomenon characterized by an inflammatory response to the presence of microorganisms or the invasion of normally sterile host tissue by those organisms.

**Bacteremia**:

The presence of viable bacteria in the blood.

**Systemic inflammatory response syndrome (SIRS):**

The systemic inflammatory response to a variety of severe clinical insults. The response is manifested by two or more of the following conditions:

(1) temperature >38^o^C or <36^o^C;

(2) heart rate >90 beats per minute;

(3) respiratory rate >20 breaths per minute or PaCO_2_, <32 mm Hg; and

(4) white blood cell count >12,000/cu mm, <4,000/cu mm, or >10% immature (band) forms

**Sepsis** :

the systemic response to infection, manifested by two or more of the following conditions as a result of infection:

1. temperature >38^o^C or <36^o^C;
2. heart rate >90 beats per minute or >110 beats per minute for children aged >12 yrs;
3. respiratory rate >20 breaths per minute or PaCO_2_, <32 mm Hg; and
4. white blood cell count >12,00Wcu mm, <4,000/cu mm, or >10% immature (band) forms or >11,00Wcu mm, <4,500/cu mm in case of children aged >12 yrs
5. hyperglycemia (>120 mg/dl) in the absence of diabetes
6. acutely Altered Mental Status with a decrease in Glasgow Coma Score ≥3 points from the baseline

**Severe sepsis**:

Sepsis induced tissue hypoperfusion or organ dysfunction reflected by any of the following evidence that is new and not explained by other known etiology of organ dysfunction

1. sepsis-induced hypotension (a systolic blood pressure <90 mm Hg or a reduction of 40 mm Hg from baseline in the absence of other causes for hypotension)
2. need for vasoactive drug to maintain BP in normal range (dopamine >5 μ*g/kg/*min or dobutamine, epinephrine at any dose)
3. any two of the following:

- unexplained metabolic acidosis: base deficit > 5.0 mEg/L
- urine output < 0.5 mL/kg/hr for more than 2 hrs despite adequate fluid resuscitation
- core to peripheral temperature gap > 3°C

1. PAO 2/ FIO 2 <300 in absence of cyanotic heart disease or preexisting lung disease
2. acute lung injury with PaO 2/FIO 2 < 200 in the presence of pneumonia as infection source
3. PaCO 2 >65 mmHg or 20 mm Hg over baseline PaCO 2
4. proven need for >50% FiO 2 to maintain saturation ≥92%
5. urine output < 0.5 mL/kg/hr for more than 2 hrs despite adequate fluid resuscitation
6. creatinine > 2.0 mg/dL (176.8 µmol/L) or 50% or 0.3 mg/dL increase in plasma creatinine above baseline level.
7. bilirubin > 2 mg/dL (34.2 µmol/L)
8. ALT 2 times upper limit of normal for age
9. platelet count < 80,000 µL
10. coagulopathy (international normalized ratio > 1.5)
11. glasgow Coma Score ≤11
12. acute change in mental status with a decrease in Glasgow Coma Score ≥3 points from abnormal baseline

**Septic shock**:

Severe sepsis associated with refractory hypotension (BP<90/60) despite adequate fluid resuscitation. Patients who are receiving inotropic or vasopressor agents may not be hypotensive at the time that perfusion abnormalities are measured and yet will be considered as septic shock.

*Adapted from*

1. *Levy MM, Fink MP, Marshall JC, et al: 2001 SCCM/ESICM/ACCP/ATS/SIS International Sepsis Definitions Conference. Crit Care Med 2003; 31:1250–1256.*
2. *The ACCP/SCCM Consensus Conference Committee; Definition of sepsis and organ failureand guidelines for the use of innovative therapies in sepsis; Chest 1992; 101: 1644-55*
3. Dellinger, R. Phillip et al, *Surviving Sepsis Campaign: International Guidelines for Management of Severe Sepsis and Septic Shock: 2012.* Critical Care Medicine, Feb.2013, Vol.41, No2.
4. Goldstein, Brahm et al, *International pediatric sepsis consensus conference: Definitions for sepsis and organ dysfunction in pediatrics.* Pediatric Critical Care Medicine, 2005, Vol.6, No.1.

**APPENDIX 1B**

**Deep vein thrombosis**

**Wells criteria**

| ***Clinical model for predicting the pre-test probability of deep-vein thrombosis. **** | |
| --- | --- |
| **Clinical Characteristic** | **Score** |
| Active cancer (patient receiving treatment for cancer within the previous 6 months or currently receiving palliative treatment) | 1 |
|  |  |
| Paralysis, paresis, or recent plaster immobilization of the lower Extremities | 1 |
|  |  |
| Recently bedridden for 3 days or more, or major surgery within the previous 12 weeks requiring general or regional anaesthesia | 1 |
|  |  |
| Localized tenderness along the distribution of the deep venous system | 1 |
|  |  |
| Entire leg swollen | 1 |
|  |  |
| Calf swelling at least 3 cm larger than that on the asymptomatic side (measured 10 cm below tibial tuberosity) | 1 |
|  |  |
| Pitting oedema confined to the symptomatic leg | 1 |
|  |  |
| Collateral superficial veins (non-varicose) | 1 |
|  |  |
| Previously documented deep-vein thrombosis | 1 |
|  |  |
| Alternative diagnosis at least as likely as deep-vein thrombosis | 1 |

*******A score of two or higher indicates that the probability of deep-vein thrombosis is likely; a score of less than two indicates that the probability of deep-vein thrombosis is unlikely. In patients with symptoms in both legs, the more symptomatic leg is used.

*Philip S. Wells et al. Evaluation of d -Dimer in the Diagnosis of Suspected Deep-Vein Thrombosis; N Engl J Med 2003;349:1227-35*

**Diagnosis of Deep Venous Thrombosis**

*Adapted from Institute for Clinical Systems Improvement. Copyright 2012. Health care guideline: venous thromboembolism diagnosis and treatment.*

**APPENDIX 1C**

**Compliance Record Form**

| **Patients** | **Often** | **Occasionally** | **Never** |
| --- | --- | --- | --- |
| This procedure causes pain or discomfort |  |  |  |
| I become anxious during the procedure |  |  |  |
| This procedure is cheap and affordable |  |  |  |
| I am satisfied with the procedure |  |  |  |
| Any specific comment | | | |
| **Nurses** | | | |
| The procedure is easy to perform |  |  |  |
| This procedure easy to learn |  |  |  |
| This procedure is time consuming |  |  |  |
| It causes frequent complication and causes harm to the patient |  |  |  |
| Patients are friendly with the procedure |  |  |  |
| Patient care is difficult during the procedure |  |  |  |
| Patients report pain or discomfort during the procedure |  |  |  |
| Resident doctors can easily solve any procedure related problem |  |  |  |
| Any specific complication or harm you observed | | | |
| **Resident Doctors** | | | |
| The procedure is easy to perform |  |  |  |
| This procedure easy to learn |  |  |  |
| This procedure is easy to monitor |  |  |  |
| Problems during the procedure is easy to handle |  |  |  |
| This procedure causes much hemodynamic instability |  |  |  |
| Nurses follow appropriate sterile techniques |  |  |  |
| Patients are comfortable during the procedure |  |  |  |
| This procedure can be applied in ward or cabin facilities |  |  |  |
| This procedure causes severe blood stream infection |  |  |  |
| Specify any particular difficulty you faced during the procedure | | | |
